# Iron chelates hitch a ride on PAT1

**DOI:** 10.1016/j.jbc.2021.100418

**Published:** 2021-03-21

**Authors:** James F. Collins

**Affiliations:** Food Science & Human Nutrition Department, University of Florida, Gainesville, Florida, USA

**Keywords:** Slc36a1, slc11a2, Dmt1, slc40a1, Fpn1, nicotianamine, iron absorption, DMT1, divalent metal-ion transporter 1, FPN1, ferroportin 1, HEPH, hephaestin, NA, nicotianamine, PAT1, proton-coupled amino acid transporter 1

## Abstract

The nicotianamine-iron chelate [NA-Fe^2+^], which is found in many plant-based foods, has been recently described as a new form of bioavailable iron in mice and chickens. How NA-Fe^2+^ is assimilated from the diet, however, remains unclear. The current investigation by Murata *et al.* has identified the proton-coupled amino acid transporter 1 (PAT1) as the main mechanism by which NA-Fe^2+^ is absorbed in the mammalian intestine. Discovery of this new form of dietary iron and elucidation of its pathway of intestinal absorption may lead to the development of improved iron supplementation approaches.

Iron is an essential nutrient for humans and other mammals with numerous critical biochemical functions; yet iron in excess is toxic. Importantly, there is no physiological means to excrete excess iron, so homeostasis must be maintained by modulating the magnitude of intestinal absorption. Dietary iron exists mainly as heme iron and nonheme (or inorganic) iron. Molecular mechanisms of nonheme iron absorption have been elucidated (1), but details of heme iron absorption remain unclear. Dietary nonheme iron exists as ferric (Fe^3+^) iron, which is insoluble in aqueous solutions. Endogenous (*e.g.*, gastric acid) and exogenous (*e.g.*, ascorbic acid) factors and enzyme action (*e.g.*, duodenal cytochrome B [DCYTB]) promote reduction of ferric iron to ferrous (Fe^2+^) iron. Fe^2+^ is then imported into duodenal enterocytes by divalent metal-ion transporter 1 (DMT1), which is required for nonheme iron absorption in mice (2) and probably in humans (3) (Fig. 1). Nonheme iron imported by DMT1 into enterocytes and iron contributed from other dietary iron sources constitute the intracellular “labile” iron pool. This iron can be utilized by the cell for metabolic purposes or exported by ferroportin 1 (FPN1). After export, Fe^2+^ is oxidized to Fe^3+^ by hephaestin (HEPH), which allows ferric iron binding to transferrin for delivery to the liver *via* the portal vein.

**Figure 1:**
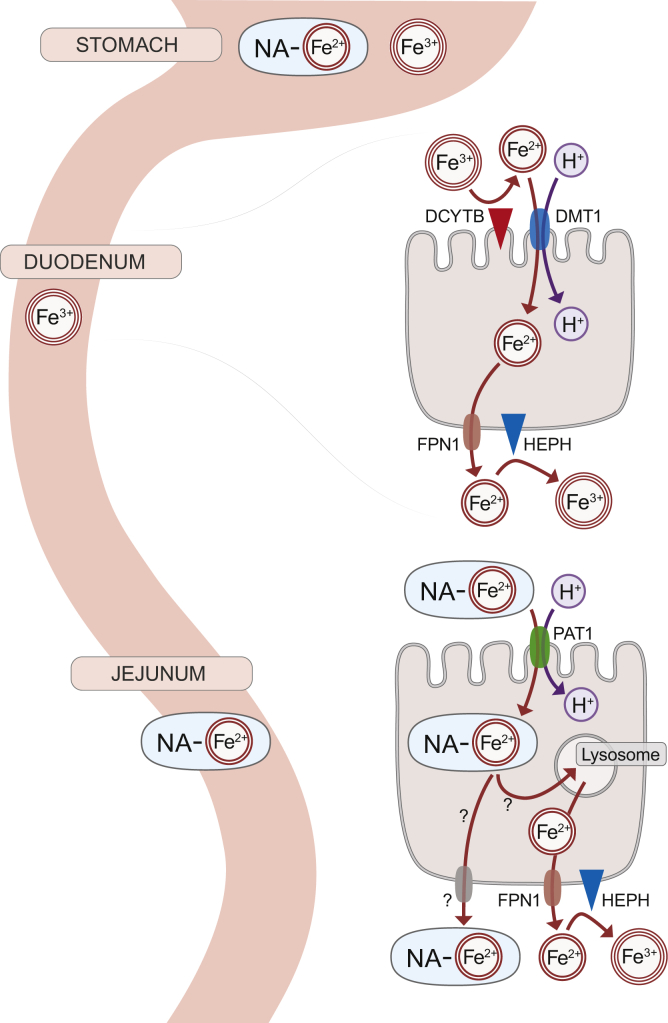
**Mechanisms of nonheme iron absorption in the duodenum and proposed mechanism of NA-Fe**^**2+**^**absorption in the proximal jejunum.** Question marks demarcate areas of uncertainty in the latter process.

Importantly, recent work has also identified another potential source of dietary iron, the nicotianamine (NA)-iron chelate [NA-Fe^2+^], derived from plant-based foods (4). The mechanism driving the absorption of this form of bioavailable iron, however, is unknown. In their recent JBC paper, Murata *et al.* have elegantly demonstrated that the NA-Fe^2+^ chelate is absorbed differently from nonheme dietary iron, advancing what is known about a fundamental biological process and perhaps providing opportunities to develop new and improved iron supplementation strategies.

NA, an iron-binding phytosiderophore found in higher plants, likely plays an innate role in defense against iron-mediated production of reactive oxygen species (5). Earlier observations demonstrated that NA-Fe^2+^ could be transported into Caco-2 cells (6) and that NA-chelated iron is bioavailable in mice (7) and chickens (8). These observations were confirmed, and expanded upon, by the recent work of Murata *et al.* (4). These authors thoughtfully noted that the NA-Fe^2+^ complex was trafficked across plant cell membranes by proteins with homology to amino acid/oligopeptide transporters in mammals. This seems logical since NA is formed from three *S*-adenosyl methionine molecules, and its structure somewhat resembles a small peptide. Thus, they tested the hypothesis that an amino acid transporter could also facilitate absorption of the NA-Fe^2+^ complex in the mammalian small intestine. Their investigation focused on proton-coupled amino acid transporter 1 (PAT1), which they viewed as a potential intestinal NA-Fe^2+^ transporter. Using radiotracer studies in Caco-2 cells (along with siRNA silencing of PAT1), functional (electrophysiological) analyses of PAT1 overexpression in *Xenopus* oocytes, and *in vivo* studies in mice, these authors demonstrated uptake of NA-^59^Fe^2+^ into the epithelium of the proximal jejunum and noted ^59^Fe appearance in the spleen, liver, and kidney. Taken together, these findings confirmed that NA-Fe^2+^ is a bioavailable source of iron *in vivo*, but most importantly, they uncovered the possible mechanism by which NA-Fe^2+^ is absorbed in the mammalian intestine (Fig. 1).

The recent investigation by Murata *et al.* expands our understanding of dietary sources of bioavailable iron and mechanisms of intestinal iron absorption. This work also raises important questions to be considered in the future. For example, is NA-Fe^2+^ targeted to lysosomes in enterocytes? If so, NA could be degraded, thus liberating the ferrous iron atom, which could be subsequently transported into the cytosol. Also, do cells that express PAT1 express the iron exporter FPN1? If so, then iron liberated from NA within cells could be exported *via* this common pathway. Or, alternatively, is the NA-Fe^2+^ complex exported from enterocytes intact, and if so, where is the iron liberated from NA? Perhaps in the liver (assuming the NA-Fe^2+^ complex is water-soluble)? The answers to these questions will shed new light on fundamental iron absorption processes crucial to whole-body iron homeostasis.

Murata *et al.*’s findings also introduce new queries concerning iron metabolism. Complex regulatory mechanisms have evolved to modulate absorption of enteral iron to match the physiological demand for iron. Historically, intestinal iron transport has been known to be influenced by the level of storage iron, erythroid demand for iron, oxygen levels, and inflammation (1). It is now clear that the liver-derived peptide hormone hepcidin is the main regulator of intestinal iron absorption, and *Hamp* (encoding hepcidin) expression in hepatocytes is modulated according to demand for dietary iron. Hepcidin modulates iron absorption by binding to FPN1 on the basolateral surface of enterocytes, causing its internalization and degradation (and thus decreasing iron efflux) (9). What remains to be elucidated is whether NA-Fe^2+^ uptake by enterocytes of the proximal jejunum is regulated according to levels of storage iron or in response to other signals that regulate absorption of nonheme iron (*e.g.*, tissue oxygen levels, erythroid demand for iron, and infection and inflammation). If PAT1 and FPN1 are coexpressed, this may indicate that NA-Fe^2+^ absorption could be regulated like absorption of nonheme iron (*i.e.*, *via* hepcidin–FPN1 interactions).

Finally, this seminal work may advance our understanding of how other essential minerals are absorbed in the intestine and could open up new avenues for treating iron deficiency in humans. As a case in point, since NA can also chelate zinc and copper, does it also facilitate uptake of these metal ions in the mammalian intestine? Also, could PAT1 facilitate the transport of iron–amino acid chelates, which are being increasingly considered as dietary iron sources with improved bioavailability (10). Additionally, perhaps NA-Fe^2+^ could be leveraged to develop a new method for iron supplementation, thus improving the options available to patients and creating new strategies that may have advantages over existing supplementation treatment regimens (*i.e.*, allowing lower dosing so as to avoid inhibition of absorption by hepcidin). Experimental consideration of these remaining issues could provide further impetus to develop NA-Fe^2+^ as a novel (and possibly improved) iron supplement.

## Conflict of interest

The authors declare that they have no conflicts of interest with the contents of this article.
